# Natural Bioactive Molecules as Potential Agents Against SARS-CoV-2

**DOI:** 10.3389/fphar.2021.702472

**Published:** 2021-08-17

**Authors:** Wei Chen, Zhihao Wang, Yawen Wang, Yiping Li

**Affiliations:** ^1^ Department of Medicinal Chemistry, School of Pharmacy, Xi’an Jiaotong University, Xi’an, China; ^2^ Biobank, First Affiliated Hospital of Xi’an Jiaotong University, Xi’an, China; ^3^ Department of Laboratory Medicine, First Affiliated Hospital of Xi’an Jiaotong University, Xi’an, China

**Keywords:** coronavirus, COVID-19, SARS-CoV-2, antiviral agents, natural bioactive molecules, therapeutic targets

## Abstract

In the past two decades, pandemics of several fatal coronaviruses have posed enormous challenges for public health, including SARS-CoV (2003), MERS-CoV (2012), and SARS-CoV-2 (2019). Among these, SARS-CoV-2 continues to ravage the world today and has lead to millions of deaths and incalculable economic damage. Till now, there is no clinically proven antiviral drug available for SARS-CoV-2. However, the bioactive molecules of natural origin, especially medicinal plants, have been proven to be potential resources in the treatment of SARS-CoV-2, acting at different stages of the viral life cycle and targeting different viral or host proteins, such as PL^pro^, 3CL^pro^, RdRp, helicase, spike, ACE2, and TMPRSS2. They provide a viable strategy to develop therapeutic agents. This review presents fundamental biological information on SARS-CoV-2, including the viral biological characteristics and invasion mechanisms. It also summarizes the reported natural bioactive molecules with anti-coronavirus properties, arranged by their different targets in the life cycle of viral infection of human cells, and discusses the prospects of these bioactive molecules for the treatment of COVID-19.

## Introduction

**FIGURE 1:**
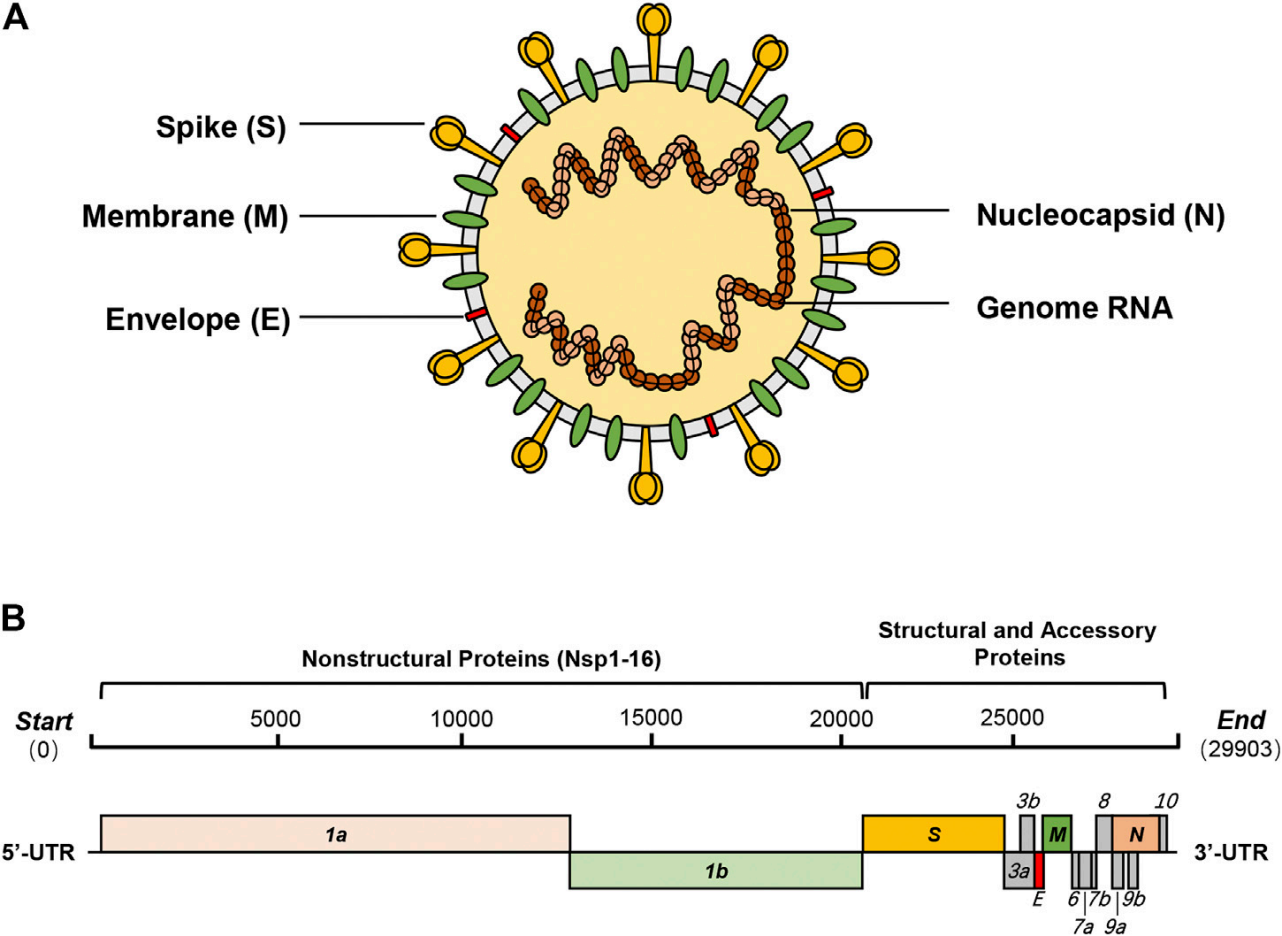
SARS-CoV-2 and its genome organization. **(A)** SARS-CoV-2 is a new β-coronavirus with a single-strand, positive-sense RNA genome, which binds with nucleocapsid proteins (N) to form the nucleocapsid. The trimeric spike glycoprotein (S) is a crucial component of virus recognition to host cells. Membrane protein (M) and envelope protein (E) play key roles in the assembly and release of virions. **(B)** SARS-CoV-2 contains a 29.9 kb genome encoding 14 ORFs. About two-thirds of the genome encodes two polyproteins (pp1a and pp1ab), which are auto-proteolytically processed into 16 non-structural proteins (Nsp1-16). The remaining one-third of the genome encodes four essential structural proteins and nine accessory proteins.

Coronaviruses, which cause respiratory tract infections in mammals and birds, are a group of enveloped, single-stranded, positive-sense RNA viruses that consist of the second-largest RNA genome (26–32 kb) only after planarian secretory cell nidovirus (PSCNV) (41.1 kb) to date (Ziebuhr, 2004; Saberi et al., 2018). Based on different antigenic cross-reactivity and genetic composition, the 26 known coronavirus species are classified into four genera (*Alphacoronavirus*, *Betacoronavirus*, *Deltacoronavirus*, and *Gammacoronavirus*)—α and β genera contain strains that are pathogenic to humans (Cleri et al., 2010). Before December 2019, six of the known coronaviruses, namely, HCoV-229E (α-CoV), HCoV-NL63 (α-CoV), HCoV-OC43 (β-CoV), HCoV-HKU1 (β-CoV), SARS-CoV (β-CoV), and MERS-CoV (β-CoV), were reported to cause diseases in humans (Arabi et al., 2017; Skariyachan et al., 2019). The first four have caused localized epidemics where patients exhibited primarily mild and self-limiting symptoms, whereas the last two can cause diseases with severe symptoms and have swept parts of the world in 2003 and 2012, respectively (Hui, 2017; Paules et al., 2020). In January 2020, another novel coronavirus, severe acute respiratory syndrome coronavirus 2 (SARS-CoV-2), was identified as the seventh coronavirus contagious to humans and had been characterized as a new member of the β-coronavirus genus (Figure 1A) (Lu et al., 2020; Wu et al., 2020).

Coronavirus disease 2019 (COVID-19) has been sweeping the world since its initial detection in Wuhan, China, in December 2019. The COVID-19 pandemic has lead to unprecedented uncertainty to modern human civilization and unforeseeable changes to global society. With more than 100 million confirmed cases and more than four million deaths, the world encounters economic contraction and the global economy declines. Many countries resorted to stringent social distancing measures to contain the spread of the virus, which included canceling group activities and limiting the movement of people. Meanwhile, numerous efforts have been devoted to SARS-CoV-2 studies, including biological characteristics, infection mechanisms, vaccine designs, and therapeutic drugs. In the foreseeable future, the continuing impacts of COVID-19 are inevitable. Anti-viral medications against SARS-CoV-2 are the key to tackling the pandemic.

New prescription medicine development is a decade-long and expensive (over $1 billion) process that consists of preclinical research, clinical trials, and commercialization (Hughes et al., 2011). Facing such challenging work, bioactive molecules of natural origin have been proven to be an excellent source for drug discovery, especially for cancer, infectious diseases, cardiovascular diseases, and multiple sclerosis (Atanasov et al., 2021). Their wide range of pharmacological activities include anti-cancer, anti-bacterial, anti-viral, anti-malaria, anti-inflammatory, anti-oxidation, anti-ageing, anti-hypertension, anti-diabetic, and immune regulation activities (Tu, 2011; Adnan et al., 2018a; Adnan et al., 2018b; Patel et al., 2019; Adnan et al., 2020; Mandadi et al., 2020; Patel et al., 2020; Siddiqui et al., 2020). In addition, the synergy of natural bioactive molecules with conventional drugs has been widely demonstrated and applied in clinical treatments (Lee et al., 2008; Sung and Lee, 2008; Russo et al., 2017). Therefore, it is a feasible strategy to identify and screen natural bioactive molecules as therapeutic agents that can effectively treat COVID-19. This review will summarize the viral biological characteristics and invasion mechanisms and highlight potential drug targets for the treatment of COVID-19. More significantly, the listed natural products with anti-CoV properties will be arranged by their different targets in the viral life cycle, mainly focusing on natural bioactive molecules with clear targets and activity data.

## Viral Biological Characteristics

### Genome Organization

As a novel β-coronavirus, the first SARS-CoV-2 genome sequence (NC_045512.2) was immediately reported in the early stage of the outbreak, which is closely related to BatCoV RaTG13 (about 96.3%), SARS-CoV (about 79%), and MERS-CoV (about 50%) (Lu et al., 2020; Paraskevis et al., 2020; Wu et al., 2020). Its 29.9 kb genome encodes as many as 14 open reading frames (ORFs), including five functional ORFs and nine putative accessory factors (Figure 1B). From 5′ to 3′, ORF1a and ORF1b occupy two-thirds of the whole genome and encode two polyproteins which are auto-proteolytically processed into 16 non-structural proteins (Nsp1-16). Then, the genome encodes four structural proteins—spike (S), envelope (E), membrane (M), and nucleocapsid (N)—with nine putative accessory factors encoded between them. Compared with SARS-CoV, the genome organization of SARS-CoV-2 shows few differences in the ORFs and Nsps. The main differences between the two are concentrated in just ORF3b, ORF8, ORF10, and spike (Chan et al., 2020; Chellapandi and Saranya, 2020; Gordon et al., 2020; Hu et al., 2020).

### Genomic Products

#### Non-structural Gene Products

Viruses express their genome products by hijacking the host’s translation machinery. The large ORF1a/b are initially translated into polyproteins (pp1a, pp1ab) and then auto-proteolytically processed into 16 non-structural proteins (Nsps) that possess specific and essential roles in the viral life cycle. Due to their almost identical sequences in many of the genomic products, the functions and roles of gene products of SARS-CoV-2 are predicted with confidence based on previous extensive studies on those of SARS-CoV. Nsp1 is predicted to be a host translation inhibitor that forms interaction with the 40S ribosomes of the host and induces host mRNA degradation (Kamitani et al., 2006; Narayanan et al., 2008; Tohya et al., 2009; Huang et al., 2011). Nsp3, known as papain-like protease (PL^pro^), is the largest multi-domain protein produced by CoVs and acts as a scaffold protein to interact with itself and to bind to other viral Nsps or host proteins; for example, Nsp3, Nsp4, and Nsp6 form a complex and are involved in viral replication (von Brunn et al., 2007; Imbert et al., 2008; Pfefferle et al., 2011; Chen et al., 2014; Ma-Lauer et al., 2016; Lei et al., 2018). Nsp5, also named main protease (M^pro^) or 3C-like protease (3CL^pro^), is a cysteine protease that can cleave the polyproteins at 11 sites and plays a vital role for the viral replication (Chou et al., 2003; Yin et al., 2007; Pfefferle et al., 2011; Chellapandi and Saranya, 2020). Interestingly, Nsp3 and Nsp5 divide the important work to complete the cleavage of the polyproteins: the former cleaves Nsp1–Nsp3, while the latter cleaves Nsp4–Nsp16 (Anand et al., 2003; Stadler et al., 2003; Prentice et al., 2004). Hence, these two proteases are considered as important targets for the design and development of anti-CoV drugs. Nsp7-Nsp8 complex acts as a primase which assists Nsp12, the viral RNA-dependent RNA polymerase (RdRp), to complete RNA synthesis, and Nsp12, as a core enzyme for the viral RNA replication, is another popular drug target against CoVs (Imbert et al., 2006; te Velthuis et al., 2010; te Velthuis et al., 2012; Xiao et al., 2012; Kirchdoerfer and Ward, 2019). Nsp13, known as NTPase/helicase, is an enzyme of the SF1 family with NTP hydrolysis activity and is translocated along with the nucleic acids by hydrolyzing ATP to retain both dsRNA and dsDNA unwinding activities; it is also considered as an attractive target for anti-CoVs (Seybert et al., 2000; Tanner et al., 2003; Ivanov et al., 2004b; Lee et al., 2010; Adedeji et al., 2012). Nsp10, a critical co-factor for activation of multiple replicative enzymes, is known to interact with both Nsp14 and Nsp16, stimulating their respective 3′-5′ exoribonuclease (ExoN) and 2′-*O*-methyltransferase activities (Decroly et al., 2008; Lugari et al., 2010; Decroly et al., 2011; Bouvet et al., 2012; Bouvet et al., 2014). In addition to the N-terminal ExoN function, the C-terminal of Nsp14 serves as N7-methyltransferase (N7Tase) (Chen et al., 2009). Nsp15, known as uridylate-specific endoribonuclease (NendoU), cooperates with Nsp14 to finish the precise cleavage of the viral RNA genome (Ivanov et al., 2004a; Bhardwaj et al., 2006; Fehr and Perlman, 2015; Xu et al., 2020).

#### Structural Gene Products

Four structural proteins, spike (S), envelope (E), membrane (M), and nucleocapsid (N), are expressed in host cells and play crucial roles in the viral infestation, assembly, and release. The S protein of SARS-CoV-2, which contains an N-terminal S1 subunit (residue 14–685) and a C-terminal S2 region (residue 686–1273), is essential for the viral infestation by binding to the same cell surface receptor of SARS-CoV, angiotensin-converting enzyme 2 (ACE2) (Hoffmann et al., 2020; Ou et al., 2020). The S1 subunit contains a receptor-binding domain (RBD), which can bind to the peptidase domain (PD) of ACE2, and shares around 70% identity with SARS-CoV. On the other hand, the S2 subunit, which helps the viral envelop fuse with the cellular membranes, shares 99% identity with SARS-CoV (Chan et al., 2020; Chellapandi and Saranya, 2020; Hu et al., 2020; Wrapp et al., 2020). Due to the essential role in viral infestation, targeting the S protein is a promising strategy for developing a drug to fight against SARS-CoV-2 (Xia et al., 2019). The small E protein plays an essential role in virus assembly and release and is implicated in the induction of host apoptosis (Liu et al., 2007; Ruch and Machamer, 2012; Schoeman and Fielding, 2019). The M protein, which is the most abundant viral constituent and acts as a scaffold protein, controls the assembly of viral particles and ensures the correct morphology of the virion (Arndt et al., 2010; Siu et al., 2014; Fung and Liu, 2019). The N protein forms the viral nucleocapsid with the RNA genome and participates in the viral RNA synthesis (Hatakeyama et al., 2008; Chang et al., 2014; McBride et al., 2014).

#### Other Gene Products

Beyond the functional proteins, the viral genome controls the expression of nine accessory proteins, which are usually regarded as dispensable for replication or structure but play other not entirely clear roles in the viral life cycle. For example, product of ORF3a is the largest accessory protein to be efficiently expressed on the cell surface and acts as an ion channel that may promote virus release (Lu et al., 2006; Michel et al., 2020). Several studies have shown that various ORF3b proteins of bat SARS-related-CoV strains have different interferon antagonistic activities. However, ORF3b of SARS-CoV-2 encodes a novel protein with no homology to ORF3b of SARS-CoV, whose function has yet to be investigated (Kopecky-Bromberg et al., 2007; Zhou et al., 2012; Chan et al., 2020). ORF8 of SARS-CoV is one of the most rapidly evolving regions among SARS-CoV genomes and is related to the viral adaptation to humans following interspecies transmission and replication, while ORF8 of SARS-CoV-2 is distant from that of known CoVs (Ceraolo and Giorgi, 2020; Chan et al., 2020; Michel et al., 2020). Overall, accessory proteins have not been adequately studied due to their dispensable roles in viral replication or structure and the fact that ORFs are short and overlapping, posing a challenge for bioinformatic prediction. However, further studies of these accessory proteins may reveal the promise of these proteins in the diagnosis, treatment, and prevention of coronaviruses because of their unique roles.

## Viral Invasion Mechanisms

**FIGURE 2:**
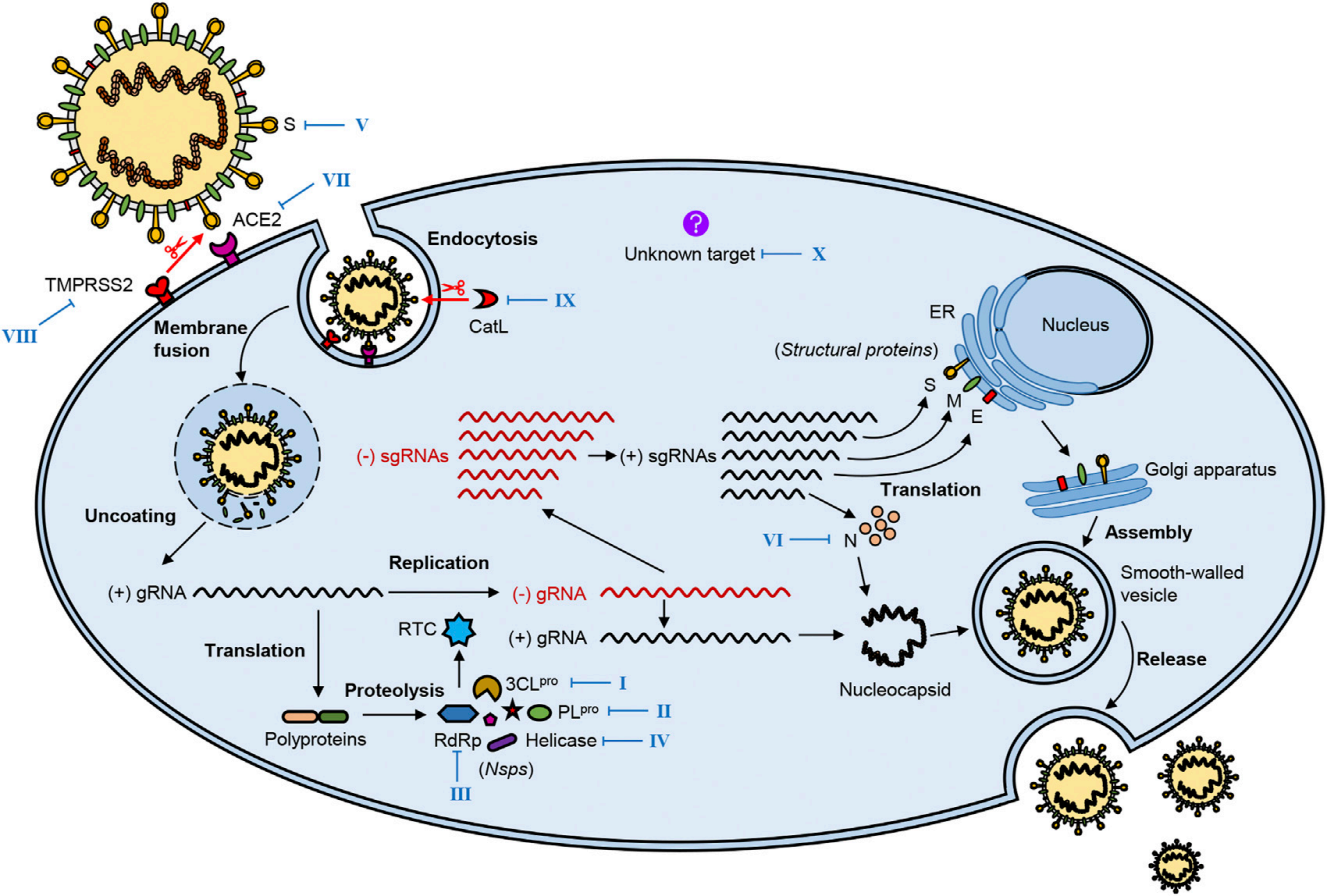
The life cycle of SARS-CoV-2 and potential therapeutic targets. SARS-CoV-2 infestation of host cells undergoes the following processes: virus attachment and entry, genome replication and transcription, virion assembly, and release. Several vital viral proteins, including 3CL^pro^, PL^pro^, RdRp, helicase, spike (S), and nucleocapsid (N), play essential roles in the life cycle of SARS-CoV-2, which may be potential therapeutic targets. Meanwhile, key host proteins significantly contribute to viral infection, such as receptor ACE2, proteases TMPRSS2, and CatL. The blue Roman numbers in the figure refer to natural bioactive molecules that inhibit different potential targets in Figures 3–8 and Tables 1–3.

After SARS-CoV-2 enters the human body, it infects the host cells mainly through these processes: virus attachment and entry, and genome replication and transcription, as well as virion assembly and release (Figure 2). These processes are accomplished through the interaction of the virus and the host cell.

### Viral Attachment and Entry

Proteolytic activation of the S protein plays a crucial role in SARS-CoV-2 attachment to and entry into host cells. The following steps complete this process: 1) recognition of the S protein and binding it to the cellular receptor; 2) alteration of the conformation and proteolysis of the S protein; 3) activation of fusion of the virion and endocytosis (Pillay, 2020).

The first step, also regarded as the beginning of SARS-CoV-2 life cycle, is the interaction of the S protein with the cell surface receptor ACE2, in which the RBD located at the S1 subunit binds to the carboxypeptidase domain of ACE2. Then, this interaction triggers a dramatic conformational change in the S2 subunit, leading to exposure and cleavage of the cleavage site at the S2 subunit which can be processed by the host cellular proteases such as cell surface transmembrane protease serine 2 (TMPRSS2) (Hoffmann et al., 2020; Matsuyama et al., 2020; Ou et al., 2020). After proteolysis of the S protein, the virion begins to fuse with the host cell membrane and enter the host cell through endocytosis.

The cleavage of the S protein is significant for SARS-CoV-2 infection and can occur at two cleavage sites processed by different proteases. The first cleavage site located at the S2 subunit can be targeted by the host cellular proteases such as TMPRSS2, which has a crucial role in activating membrane fusion between the virus and the host cell. Similarly, TMPRSS4, another serine protease in the same family, plays a similar role to TMPRSS2 in SARS-CoV-2 infection. Furthermore, recent studies have shown that camostat mesylate, a selective inhibitor of TMPRSS, can inhibit SARS-CoV-2 infection (Hoffmann et al., 2020; Zang et al., 2020). In addition, some other host cell proteases, such as cathepsin L (CatL), can also proteolytically activate the S protein of SARS-CoV-2 and then initiate the process of cellular entry (Ou et al., 2020). The second cleavage site is the furin cleavage site (Arg-Arg-Ala-Arg) between the S1 and S2 domains, common to other human CoVs like MERS-CoV but interestingly absent from SARS-CoV. The furin cleavage site can reduce the stability of the S protein and facilitate the conformational change required for RBD exposure and the subsequent binding to ACE2. Furin-like proteases are widely expressed in various cell types, especially in the respiratory tract, so the presence of the furin cleavage site in the S protein is thought to increase the infectivity of SARS-CoV-2 or alter its pathogenicity (Walls et al., 2020; Wrobel et al., 2020).

### Genome Replication and Transcription

After the completion of virus attachment and entry, the nucleocapsid is released into the host cytoplasm; then, virus replication is initiated in the cytoplasm. The virus hijacks the ribosome of the host cell; this is followed by the translation and auto-proteolysis of the polyproteins pp1a and pp1ab into 16 Nsps, which altogether form the replicase-transcriptase complex (RTC) that controls the processes of replication and translation. Mediated by RTC, the viral genomic RNA is replicated to full-length negative-sense (−)RNA; then, the (−)RNA is used as a template to synthesize new genomic (+)RNA and a series of different sgRNAs, the latter of which are translated into viral structural and accessory proteins (Ziebuhr, 2005; Masters, 2006).

### Virion Assembly and Release

When the base components are prepared, the virion assembly follows. First, the membrane-bound structural proteins, E, M, and S, are inserted into the endoplasmic reticulum (ER) and then transported to the ER-Golgi intermediate compartment (ERGIC). The N protein wraps the new genomic RNA to form a nucleocapsid, which then transits to ERGIC. The nucleocapsid and membrane-bound components coalesce to assemble virion mediated by the M protein in ERGIC. Finally, progeny virions are transported to the plasma membrane in smooth-walled vesicles and released by exocytosis (Masters, 2006; Fung and Liu, 2019; Russo et al., 2020).

## Anti-CoV Bioactive Molecules Targeting Different Proteins

In the previous sections, we have described fundamental biological information of SARS-CoV-2, the vast majority of which is conserved among other known coronaviruses especially SARS-CoV. In this dire SARS-CoV-2 pandemic with no effective drug, screening natural bioactive molecules from natural products with known anti-CoV activity can significantly accelerate the development of effective drugs against SARS-CoV-2. In this section, we will summarize natural bioactive molecules that have been reported to exhibit anti-CoV activity targeting different vital proteins, including several crucial viral and host proteins. As natural agents against SARS-CoV are the most widely reported, we will mainly focus on natural bioactive molecules found in SARS-CoV studies and introduce a few natural inhibitors against MERS-CoV or SARS-CoV-2. The description of these natural bioactive molecules will be developed according to their different targets.

### Viral Proteins

#### Viral Proteases

**FIGURE 3:**
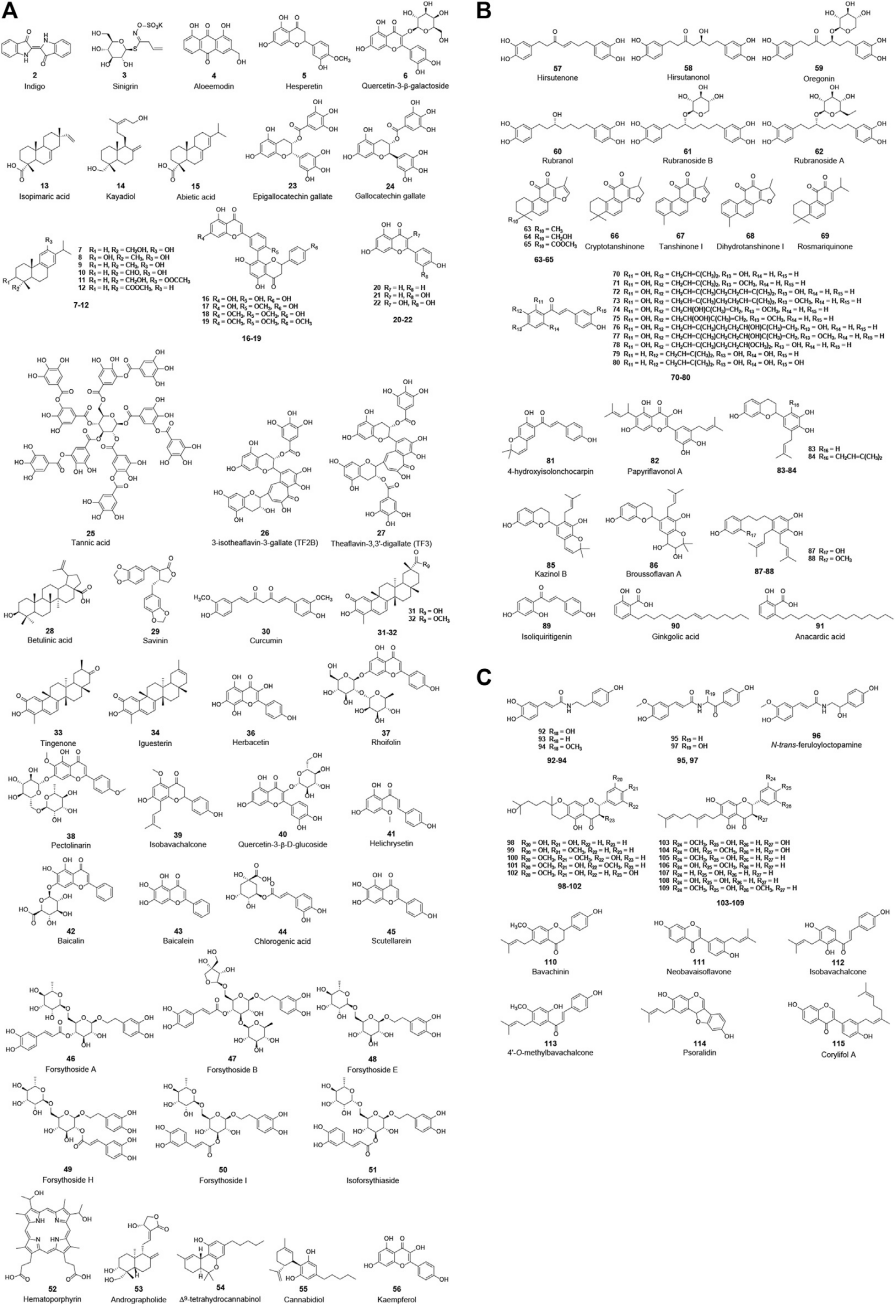
Chemical structure of different natural compounds targeting **(A)** 3CL^pro^ (**I**), **(B)** both 3CL^pro^ and PL^pro^, and **(C)** PL^pro^ (**II**). Quercetin-3-β-galactoside (**6**), quercetin (**22**), curcumin (**31**), and kaempferol (**57**) inhibit both 3CL^pro^ and PL^pro^ but are not repeatedly displayed in **(B)**.

During the replication of the virus, PL^pro^ and 3CL^pro^ are responsible for the cleavage of the polyproteins; as a result, they are considered as the most popular targets for the design and development of anti-CoV drugs. Many synthetic compounds targeting these proteases have been reported, such as rupintrivir, lopinavir, and ritonavir. Due to the inherent peptidase activity, a lot of work has been done in designing peptidomimetic inhibitors for these proteases, which will not be discussed here in detail (Hu et al., 2020; Christy et al., 2021). Furthermore, many natural bioactive molecules, mostly flavonoids, have also been shown to inhibit PL^pro^ and 3CL^pro^ (Figure 3; Table 1).

**TABLE 1:** Natural compounds targeting viral proteins: 3CL^pro^ (**I**), PL^pro^ (**II**), RdRp (**III**), NTPase/helicase (**IV**), S protein (**V**), and N protein (**VI**).

No	Viral strain	Compound	IC_50_/EC_50_	Target	References
1	SARS-CoV	*Isatis tinctoria* L. root extract	53.80 μg/ml	3CL^pro^	Lin et al. (2005)
2	SARS-CoV	Indigo	300.00 μM	3CL^pro^	Lin et al. (2005)
3	SARS-CoV	Sinigrin	121.00 μM	3CL^pro^	Lin et al. (2005)
4	SARS-CoV	Aloe emodin	132.00 μM	3CL^pro^	Lin et al. (2005)
5	SARS-CoV	Hesperetin	60.00 μM	3CL^pro^	Lin et al. (2005)
6	SARS-CoV	Quercetin-3-β-galactoside	42.79 μM	3CL^pro^	Chen et al. (2006)
7	SARS-CoV	18-Hydroxyferruginol	45.80 μM	3CL^pro^	Ryu et al. (2010a)
8	SARS-CoV	Honokiol	39.10 μM	3CL^pro^	Ryu et al. (2010a)
9	SARS-CoV	Ferruginol	92.70 μM	3CL^pro^	Ryu et al. (2010a)
10	SARS-CoV	18-Oxoferruginol	70.50 μM	3CL^pro^	Ryu et al. (2010a)
11	SARS-CoV	*O*-acetyl-18-hydroxyferruginol	78.60 μM	3CL^pro^	Ryu et al. (2010a)
12	SARS-CoV	Methyl dehydroabietate	46.70 μM	3CL^pro^	Ryu et al. (2010a)
13	SARS-CoV	Isopimaric acid	28.90 μM	3CL^pro^	Ryu et al. (2010a)
14	SARS-CoV	Kayadiol	75.20 μM	3CL^pro^	Ryu et al. (2010a)
15	SARS-CoV	Abietic acid	58.00 μM	3CL^pro^	Ryu et al. (2010a)
16	SARS-CoV	Amentoflavone	8.30 μM	3CL^pro^	Ryu et al. (2010a)
17	SARS-CoV	Bilobetin	72.30 μM	3CL^pro^	Ryu et al. (2010a)
18	SARS-CoV	Ginkgetin	32.00 μM	3CL^pro^	Ryu et al. (2010a)
19	SARS-CoV	Sciadopitysin	38.40 μM	3CL^pro^	Ryu et al. (2010a)
20	SARS-CoV	Apigenin	280.80 μM	3CL^pro^	Ryu et al. (2010a)
21	SARS-CoV	Luteolin	20.20 μM	3CL^pro^	Ryu et al. (2010a)
22	SARS-CoV	Quercetin	23.80 μM	3CL^pro^	Ryu et al. (2010a)
22	SARS-CoV	Quercetin	73.00 μM	3CL^pro^	Nguyen et al. (2012)
23	SARS-CoV	Epigallocatechin gallate	73.00 μM	3CL^pro^	Nguyen et al. (2012)
24	SARS-CoV	Gallocatechin gallate	47.00 μM	3CL^pro^	Nguyen et al. (2012)
25	SARS-CoV	Tannic acid	3.00 μM	3CL^pro^	Chen et al. (2005)
26	SARS-CoV	3-Isotheaflavin-3-gallate (TF2B)	7.00 μM	3CL^pro^	Chen et al. (2005)
27	SARS-CoV	Theaflavin-3,3′-digallate (TF3)	9.50 μM	3CL^pro^	Chen et al. (2005)
28	SARS-CoV	Betulinic acid	10.00 μM	3CL^pro^	Wen et al. (2007)
29	SARS-CoV	Savinin	25.00 μM	3CL^pro^	Wen et al. (2007)
30	SARS-CoV	Curcumin	40.00 μM	3CL^pro^	Wen et al. (2007)
30	SARS-CoV	Curcumin	23.50 μM	3CL^pro^	Ryu et al. (2010b)
31	SARS-CoV	Celastrol	10.30 μM	3CL^pro^	Ryu et al. (2010b)
32	SARS-CoV	Pristimererin	5.50 μM	3CL^pro^	Ryu et al. (2010b)
33	SARS-CoV	Tingenone	9.90 μM	3CL^pro^	Ryu et al. (2010b)
34	SARS-CoV	Iguesterin	2.60 μM	3CL^pro^	Ryu et al. (2010b)
35	SARS-CoV	*Rheum palmatum* L. extract (RH121)	13.76 μg/ml	3CL^pro^	Luo et al. (2009)
36	SARS-CoV	Herbacetin	33.17 μM	3CL^pro^	Jo et al. (2020)
37	SARS-CoV	Rhoifolin	27.45 μM	3CL^pro^	Jo et al. (2020)
38	SARS-CoV	Pectolinarin	37.78 μM	3CL^pro^	Jo et al. (2020)
36	MERS-CoV	Herbacetin	40.59 μM	3CL^pro^	Jo et al. (2019)
39	MERS-CoV	Isobavachalcone	35.85 μM	3CL^pro^	Jo et al. (2019)
40	MERS-CoV	Quercetin-3-β-D-glucoside	37.03 μM	3CL^pro^	Jo et al. (2019)
41	MERS-CoV	Helichrysetin	67.04 μM	3CL^pro^	Jo et al. (2019)
22	SARS-CoV-2	Quercetin	K_i_ = 7.00 μM	3CL^pro^	Abian et al. (2020)
42	SARS-CoV-2	Baicalin	6.41 μM	3CL^pro^	Su et al. (2020)
43	SARS-CoV-2	Baicalein	0.94 μM	3CL^pro^	Su et al. (2020)
44	SARS-CoV-2	Chlorogenic acid	39.48 μM	3CL^pro^	Su et al. (2020)
45	SARS-CoV-2	Scutellarein	3.02 μM	3CL^pro^	Su et al. (2020)
46	SARS-CoV-2	Forsythoside A	3.18 μM	3CL^pro^	Su et al. (2020)
47	SARS-CoV-2	Forsythoside B	2.88 μM	3CL^pro^	Su et al. (2020)
48	SARS-CoV-2	Forsythoside E	6.88 μM	3CL^pro^	Su et al. (2020)
49	SARS-CoV-2	Forsythoside H	10.17 μM	3CL^pro^	Su et al. (2020)
50	SARS-CoV-2	Forsythoside I	5.47 μM	3CL^pro^	Su et al. (2020)
51	SARS-CoV-2	Isoforsythiaside	5.85 μM	3CL^pro^	Su et al. (2020)
25	SARS-CoV-2	Tannic acid	13.40 μM	3CL^pro^	Wang et al. (2020)
25	SARS-CoV-2	Tannic acid	2.10 μM	3CL^pro^	Coelho et al. (2020)
52	SARS-CoV-2	Hematoporphyrin	3.90 μM	3CL^pro^	Coelho et al. (2020)
53	SARS-CoV-2	Andrographolide	15.05 μM	3CL^pro^	Shi et al. (2020)
54	SARS-CoV-2	Δ^9^-Tetrahydrocannabinol	10.25 μM (antiviral activity)	3CL^pro^	Raj et al. (2021)
55	SARS-CoV-2	Cannabidiol	7.91 μM (antiviral activity)	3CL^pro^	Raj et al. (2021)
56	SARS-CoV-2	Kaempferol	34.46 μM (antiviral activity)	3CL^pro^	Khan et al. (2021)
57	SARS-CoV	Hirsutenone	36.20 μM	3CL^pro^	Park et al. (2012b)
			4.10 μM	PL^pro^	
58	SARS-CoV	Hirsutanonol	105.60 μM	3CL^pro^	Park et al. (2012b)
			7.80 μM	PL^pro^	
59	SARS-CoV	Oregonin	129.50 μM	3CL^pro^	Park et al. (2012b)
			20.10 μM	PL^pro^	
60	SARS-CoV	Rubranol	144.60 μM	3CL^pro^	Park et al. (2012b)
			12.30 μM	PL^pro^	
61	SARS-CoV	Rubranoside B	105.30 μM	3CL^pro^	Park et al. (2012b)
			8.00 μM	PL^pro^	
62	SARS-CoV	Rubranoside A	102.10 μM	3CL^pro^	Park et al. (2012b)
			9.10 μM	PL^pro^	
30	SARS-CoV	Curcumin	5.70 μM	PL^pro^	Park et al. (2012b)
63	SARS-CoV	Tanshinone IIA	89.10 μM	3CL^pro^	Park et al. (2012c)
			1.60 μM	PL^pro^	
64	SARS-CoV	Tanshinone IIB	24.80 μM	3CL^pro^	Park et al. (2012c)
			10.70 μM	PL^pro^	
65	SARS-CoV	Methyl tanshinonate	21.10 μM	3CL^pro^	Park et al. (2012c)
			9.20 μM	PL^pro^	
66	SARS-CoV	Cryptotanshinone	226.70 μM	3CL^pro^	Park et al. (2012c)
			0.80 μM	PL^pro^	
67	SARS-CoV	Tanshinone I	38.70 μM	3CL^pro^	Park et al. (2012c)
			8.80 μM	PL^pro^	
68	SARS-CoV	Dihydrotanshinone I	14.40 μM	3CL^pro^	Park et al. (2012c)
			4.90 μM	PL^pro^	
69	SARS-CoV	Rosmariquinone	21.10 μM	3CL^pro^	Park et al. (2012c)
			30.00 μM	PL^pro^	
70	SARS-CoV	Isobavachalcone	39.40 μM	3CL^pro^	Park et al. (2016)
			13.00 μM	PL^pro^	
71	SARS-CoV	4-Hydroxyderricin	81.40 μM	3CL^pro^	Park et al. (2016)
			26.00 μM	PL^pro^	
72	SARS-CoV	Xanthoangelol	38.40 μM	3CL^pro^	Park et al. (2016)
			11.70 μM	PL^pro^	
73	SARS-CoV	Xanthoangelol F	34.10 μM	3CL^pro^	Park et al. (2016)
			5.60 μM	PL^pro^	
74	SARS-CoV	Xanthoangelol D	26.60 μM	3CL^pro^	Park et al. (2016)
			19.30 μM	PL^pro^	
75	SARS-CoV	Xanthoangelol E	11.40 μM	3CL^pro^	Park et al. (2016)
			1.20 μM	PL^pro^	
76	SARS-CoV	Xanthoangelol B	22.20 μM	3CL^pro^	Park et al. (2016)
			11.70 μM	PL^pro^	
77	SARS-CoV	Xanthoangelol G	129.80 μM	3CL^pro^	Park et al. (2016)
			46.40 μM	PL^pro^	
78	SARS-CoV	Xanthokeistal A	44.10 μM	3CL^pro^	Park et al. (2016)
			21.10 μM	PL^pro^	
79	SARS-CoV	Broussochalcone B	57.80 μM	3CL^pro^	Park et al. (2017)
			11.60 μM	PL^pro^	
80	SARS-CoV	Broussochalcone A	88.10 μM	3CL^pro^	Park et al. (2017)
			9.20 μM	PL^pro^	
81	SARS-CoV	4-Hydroxyisolonchocarpin	202.70 μM	3CL^pro^	Park et al. (2017)
			35.40 μM	PL^pro^	
82	SARS-CoV	Papyriflavonol A	103.60 μM	3CL^pro^	Park et al. (2017)
			3.70 μM	PL^pro^	
83	SARS-CoV	3’-(3-Methylbut-2-enyl)-3′,4,7-trihydroxyflavane	30.20 μM	3CL^pro^	Park et al. (2017)
			35.80 μM	PL^pro^	
84	SARS-CoV	Kazinol A	84.80 μM	3CL^pro^	Park et al. (2017)
			66.20 μM	PL^pro^	
85	SARS-CoV	Kazinol B	233.30 μM	3CL^pro^	Park et al. (2017)
			31.40 μM	PL^pro^	
86	SARS-CoV	Broussoflavan A	92.40 μM	3CL^pro^	Park et al. (2017)
			30.40 μM	PL^pro^	
87	SARS-CoV	Kazinol F	43.30 μM	3CL^pro^	Park et al. (2017)
			27.80 μM	PL^pro^	
88	SARS-CoV	Kazinol J	64.20 μM	3CL^pro^	Park et al. (2017)
			15.20 μM	PL^pro^	
89	SARS-CoV	Isoliquiritigenin	61.90 μM	3CL^pro^	Park et al. (2017)
			24.60 μM	PL^pro^	
56	SARS-CoV	Kaempferol	116.30 μM	3CL^pro^	Park et al. (2017)
			16.30 μM	PL^pro^	
22	SARS-CoV	Quercetin	52.70 μM	3CL^pro^	Park et al. (2017)
			8.60 μM	PL^pro^	
6	SARS-CoV	Quercetin-3-β-galactoside	128.80 μM	3CL^pro^	Park et al. (2017)
			51.90 μM	PL^pro^	
90	SARS-CoV-2	Ginkgolic acid	1.79 μM	3CL^pro^	Chen et al. (2021)
			16.30 μM	PL^pro^	
91	SARS-CoV-2	Anacardic acid	2.07 μM	3CL^pro^	Chen et al. (2021)
			17.08 μM	PL^pro^	
92	SARS-CoV	*N*-*trans*-caffeoyltyramine	44.40 μM	PL^pro^	Song et al. (2014)
93	SARS-CoV	*N-trans*-coumaroyltyramine	38.80 μM	PL^pro^	Song et al. (2014)
94	SARS-CoV	*N-trans*-feruloyltyramine	70.10 μM	PL^pro^	Song et al. (2014)
95	SARS-CoV	Terrestriamide	21.50 μM	PL^pro^	Song et al. (2014)
96	SARS-CoV	*N-trans*-feruloyloctopamine	26.60 μM	PL^pro^	Song et al. (2014)
97	SARS-CoV	Terrestrimine	15.80 μM	PL^pro^	Song et al. (2014)
98	SARS-CoV	Tomentin A	6.20 μM	PL^pro^	Cho et al. (2013)
99	SARS-CoV	Tomentin B	6.10 μM	PL^pro^	Cho et al. (2013)
100	SARS-CoV	Tomentin C	11.60 μM	PL^pro^	Cho et al. (2013)
101	SARS-CoV	Tomentin D	12.50 μM	PL^pro^	Cho et al. (2013)
102	SARS-CoV	Tomentin E	5.00 μM	PL^pro^	Cho et al. (2013)
103	SARS-CoV	3′-*O*-methyldiplacol	9.50 μM	PL^pro^	Cho et al. (2013)
104	SARS-CoV	4′-*O*-methyldiplacol	9.20 μM	PL^pro^	Cho et al. (2013)
105	SARS-CoV	3′-*O*-methyldiplacone	13.20 μM	PL^pro^	Cho et al. (2013)
106	SARS-CoV	4′-*O*-methyldiplacone	12.70 μM	PL^pro^	Cho et al. (2013)
107	SARS-CoV	Mimulone	14.40 μM	PL^pro^	Cho et al. (2013)
108	SARS-CoV	Diplacone	10.40 μM	PL^pro^	Cho et al. (2013)
109	SARS-CoV	6-Geranyl-4′,5,7-trihydroxy-3′,5′-dimethoxyflavanone	13.90 μM	PL^pro^	Cho et al. (2013)
110	SARS-CoV	Bavachinin	38.40 μM	PL^pro^	Kim et al. (2014)
111	SARS-CoV	Neobavaisoflavone	18.30 μM	PL^pro^	Kim et al. (2014)
112	SARS-CoV	Isobavachalcone	7.30 μM	PL^pro^	Kim et al. (2014)
113	SARS-CoV	4′-*O*-methylbavachalcone	10.10 μM	PL^pro^	Kim et al. (2014)
114	SARS-CoV	Psoralidin	4.20 μM	PL^pro^	Kim et al. (2014)
115	SARS-CoV	Corylifol A	32.20 μM	PL^pro^	Kim et al. (2014)
116	SARS-CoV	Kwan du Bu Fei Dang exact	471.30 μg/ml	RdRp	Fung et al. (2011)
117	SARS-CoV	*Houttuynia cordata* exact	251.10 μg/ml	RdRp	Fung et al. (2011)
118	SARS-CoV	*Ganoderma lucidum* exact	41.90 μg/ml	RdRp	Fung et al. (2011)
119	SARS-CoV	*Coriolus versicolor* exact	108.40 μg/ml	RdRp	Fung et al. (2011)
120	SARS-CoV	*Sinomenium acutum* exact	198.60 μg/ml	RdRp	Fung et al. (2011)
22	SARS-CoV	Quercetin	8.10 μM	NTPase/helicase	Lee et al. (2009b)
121	SARS-CoV	Myricetin	2.71 μM	NTPase/helicase	Yu et al. (2012)
122	SARS-CoV	Scutellarein	0.86 μM	NTPase/helicase	Yu et al. (2012)
123	SARS-CoV	Tetra-*O*-galloyl-β-d-glucose (TGG)	4.50 μM (antiviral activity)	S protein	Yi et al. (2004)
21	SARS-CoV	Luteolin	10.60 μM (antiviral activity)	S protein	Yi et al. (2004)
124	SARS-CoV	Emodin	200.00 μM	S protein	Ho et al. (2007)
125	SARS-CoV	Griffithsin	48–94 nM (antiviral activity)	S protein	O'Keefe et al. (2010)
126	SARS-CoV	*Urtica dioica* L. agglutinin (UDA)	0.60–2.60 μg/ml (antiviral activity)	S protein	Kumaki et al. (2011)
127	SARS-CoV-2	Kobophenol A	1.81 μM	S protein	Gangadevi et al. (2021)
128	SARS-CoV	(-)-Catechin gallate	0.05 μg/ml	N protein	Roh (2012)
24	SARS-CoV	(-)-Gallocatechin gallate	0.05 μg/ml	N protein	Roh (2012)

Among the natural products studied for their activity against SARS-CoV, the largest number of bioactive molecules has been reported to have 3CL^pro^ inhibitory activity (**I**, Figure 3A). Lin et al. used cell-free and cell-based cleavage assays to study anti-SARS-CoV 3CL^pro^ activities of *Isatis tinctoria* L. root extract, five major compounds of *Isatis tinctoria* L. root, and seven plant-derived phenolic compounds. Their study showed that *Isatis tinctoria* L. root extract (**1**), indigo (**2**), sinigrin (**3**), aloe emodin (**4**), and hesperetin (**5**) exhibited significant inhibitory activity against SARS-CoV 3CL^pro^ in the micromolar range. In particular, hesperetin showed the best activity among these compounds and dose-dependently inhibited cleavage activity of SARS-CoV 3CL^pro^ with IC_50_ values of 60.00 and 8.30 μM in cell-free and cell-based cleavage assays, respectively (Lin et al., 2005). Interestingly, although quercetin was reported to have anti-SARS-CoV activity, it did not show anti-3CL^pro^ activity in this study (Yi et al., 2004; Lin et al., 2005). However, in several subsequent studies, quercetin showed inhibitory activity against SARS-CoV 3CL^pro^ or was used as a positive control. A natural glycoside derivative of quercetin, quercetin-3-β-galactoside (**6**), was shown to block the cleavage activity of SARS-CoV 3CL^pro^ with an IC_50_ of 42.76 μM. Through molecular modeling and Q189A mutation of 3CL^pro^, Gln189 was identified as an important amino acid residue that played a vital role in quercetin-3-β-galactoside binding to 3CL^pro^. The Q186A mutation did not change the enzymatic activity of 3CL^pro^, while the SPR and FRET assay results showed that both the binding affinity and the inhibitory potency of quercetin-3-β-galactoside to the mutated 3CL^pro^ were significantly lower than those to the wild-type 3CL^pro^ (Chen et al., 2006). Ryu et al. implemented FRET assay to evaluate the anti-SARS-CoV 3CL^pro^ activity of 12 compounds extracted from *Torreya nucifera* (L.) Siebold & Zucc., including eight diterpenoids and four biflavonoids, and abietic acid (**15**, IC_50_ = 58.00 μM), apigenin (**20**, IC_50_ = 280.80 μM), luteolin (**21**, IC_50_ = 20.20 μM), and quercetin (**22**, IC_50_ = 23.80 μM) were used as positive control compounds. Among these 12 compounds, the biflavone amentoflavone (**16**) showed the most potent 3CL^pro^ inhibitory effect with an IC_50_ of 8.30 μM (Ryu et al., 2010a). In another study, the anti-3CL^pro^ activities of seven flavonoid compounds were evaluated by *in vitro* 3CL^pro^ inhibition and kinetic assays, among which quercetin (**22**), epigallocatechin gallate (**23**), and gallocatechin gallate (**24**) showed inhibitory effects on SARS-CoV 3CL^pro^ with IC_50_ values of 73.00, 73.00, and 47.00 μM, respectively (Nguyen et al., 2012). Through screening a natural product library consisting of 720 compounds and evaluating extracts of several types of tea, including green tea, oolong tea, Puer tea, and black tea, three natural products—tannic acid (**25**, IC_50_ = 3.00 μM), 3-isotheaflavin-3-gallate (**26**, IC_50_ = 7.00 μM), and theaflavin-3, 3′-digallate (**27**, IC_50_ = 9.50 μM)—were found to be SARS-CoV 3CL^pro^ inhibitors (Chen et al., 2005). Wen et al. evaluated the anti-SARS-CoV activity of 221 phytocompounds using a cell-based assay measuring SARS-CoV-induced cytopathogenic effect on Vero E6 cells and found that 22 compounds were potent inhibitors at concentrations between 3.30 and 10.00 µM. Of these, betulinic acid (**28**), savinin (**29**), and curcumin (**30**) displayed potent inhibition toward 3CL^pro^ with IC_50_ values of 10.00, 25.00, and 40.00 µM, respectively, and the first two blocked the cleavage activity of the 3CL^pro^ by competitive inhibition (Wen et al., 2007). Curcumin (**30**, IC_50_ = 23.50 μM) was used as a positive control in another study which reported that four quinone-methide triterpene derivatives isolated from *Tripterygium wilfordii* Hook. f., namely, celastrol (**31**), pristimerin (**32**), tingenone (**33**), and iguesterin (**34**), were identified as inhibitors of SARS-CoV 3CL^pro^ (Ryu et al., 2010b). Luo et al. reported that several components derived from *Rheum palmatum* L. showed high inhibitory activity against SARS-CoV 3CL^pro^ in *in vitro* assay. The most active among them, RH121 (**35**), had an IC_50_ of 13.76 μg/ml, and the inhibition rate was up to 96% (Luo et al., 2009). Jo et al. applied a flavonoid library to screen and identify herbacetin (**36**), rhoifolin (**37**), and pectolinarin (**38**) as prominent inhibitors blocking the activity of SARS-CoV 3CL^pro^ with IC_50_ values of 33.17, 27.45, and 37.78 μM, respectively (Jo et al., 2020). In addition, the same author reported that herbacetin (**36**), isobavachalcone (**39**), quercetin-3-β-D-glucoside (**40**), and helichrysetin (**41**) were inhibitors against MERS-CoV 3CL^pro^ with IC_50_ values of 40.59, 35.85, 37.03, and 67.04 μM, respectively (Jo et al., 2019).

With the SARS-CoV-2 outbreak, a lot of effort has been devoted to the discovery of natural bioactive molecules against SARS-CoV-2. Quercetin (**22**), a well-known flavonoid reported as an anti-SARS-CoV natural product, was identified to inhibit 3CL^pro^ of SARS-CoV-2 with an inhibition constant K_i_ of 7.00 μM in an experimental screening of a small chemical library (Abian et al., 2020). Shuanghuanglian preparation is a traditional Chinese medicine with a long history in treating respiratory tract infection in China, and it received widespread attention after the SARS-CoV-2 pandemic. Su et al. recently reported that the oral liquid of Shuanghuanglian, the lyophilized powder of Shuanghuanglian for injection, and their bioactive components exhibited dose-dependent inhibition against the SARS-CoV-2 3CL^pro^ and the replication of SARS-CoV-2 in Vero E6 cells. Among these bioactive components, baicalin (**42**) and baicalein (**43**) were identified as the first non-covalent and non-peptidomimetic inhibitors of SARS-CoV-2 3CL^pro^, which blocked the cleavage activity of SARS-CoV-2 3CL^pro^ with IC_50_ values of 6.41 and 0.94 μM, as well as showing potent antiviral activities in a cell-based system. Furthermore, the crystal complex structure of SARS-CoV-2 3CL^pro^ and baicalein showed that this small flavonoid occupied the core substrate-binding pocket by interacting with two catalytic residues, the crucial S1/S2 subsites and the oxyanion loop, thereby blocking the activity of 3CL^pro^ by competitive inhibition (Su et al., 2020). Tannic acid (**25**) was recently reported to directly interact with SARS-CoV-2 3CL^pro^ with a dissociation constant (K_D_) of 1.10 μM and inhibited 3CL^pro^ with an IC_50_ of 13.40 μM (Wang et al., 2020). Additionally, a similar observation of tannic acid (**25**) with anti-SARS-CoV-2 3CL^pro^ activity (IC_50_ = 2.10 μM) was repeatedly reported in another study that also identified hematoporphyrin (**52**, IC_50_ = 3.90 μM) as a potent inhibitor against SARS-CoV-2 3CL^pro^ (Coelho et al., 2020). Andrographolide (**53**), a lactone diterpenoid compound highly abundant in leaves of *Andrographis paniculata* (Burm. f.) Nees, was reported to suppress 3CL^pro^ activities of both SARS-CoV and SARS-CoV-2 with IC_50_ values of 5.00 and 15.05 μM. Mass spectrometry (MS) and molecular modeling analysis suggested that andrographolide formed a covalent bond with the active site Cys145 and occupied the catalytic pockets of both viral 3CL^pro^s (Shi et al., 2020). In addition, Raj et al. used *in silico* and *in vitro* experiments to determine anti-SARS-CoV-2 activities of a series of cannabinoids (CBDs) and identified Δ^9^-tetrahydrocannabinol (**54**) and cannabidiol (**55**) as effective agents against SARS-CoV-2 with IC_50_ values of 10.25 and 7.91 μM. Molecular dynamic simulation and density functional theory showed the two compounds formed stable conformations with the active binding pocket of SARS-CoV-2 3CL^pro^ (Raj et al., 2021). Khan et al. employed similar approaches and reported that kaempferol (**56**) had an anti-SARS-CoV-2 activity with an IC_50_ value of 34.46 μM in *in vitro* assay and targeted SARS-CoV-2 3CL^pro^ (Khan et al., 2021).

Another protease, PL^pro^, is also regarded as an ideal anti-CoV drug target, and a lot of natural inhibitors targeting this protease have been reported (**II**, Figure 2). Among these bioactive molecules, some have inhibitory activity against both PL^pro^ and 3CL^pro^, although most are also somewhat selective (Figure 3B). Park et al. published several excellent articles reporting a range of natural bioactive molecules that inhibited both PL^pro^ and 3CL^pro^ (Park et al., 2012b; Park et al., 2012c; Park et al., 2016; Park et al., 2017). In 2012, they reported nine diarylheptanoids from *Alnus japonica* (Thunb.) Steud. and evaluated their inhibitory activities against SARS-CoV PL^pro^ and 3CL^pro^ using *in vitro* assays, and six of these compounds selectively exhibited stronger inhibitory activities against PL^pro^ than 3CL^pro^. Hirsutenone (**57**) displayed the most potent PL^pro^ inhibitory activity with an IC_50_ value of 4.10 μM, similar to positive control curcumin (**30**, IC_50_ = 5.70 μM) (Park et al., 2012b). They reported that seven tanshinones derived from *Salvia miltiorrhiza* Bunge exhibited excellent inhibitory activities against both PL^pro^ and 3CL^pro^ of SARS-CoV in the same year. Nevertheless, these extract components showed stronger activities against PL^pro^ than 3CL^pro^, of which cryptotanshinone (**66**) had the lowest IC_50_ value of 0.80 μM against SARS-CoV PL^pro^ (Park et al., 2012c). Using cell-free and cell-based assays, the inhibitory activities of 13 constituents from *Angelica keiskei* (Miq.) Koidz. against SARS-CoV proteases were determined, which showed that chalcones were potent inhibitors against PL^pro^ and 3CL^pro^ of SARS-CoV. Among them, xanthoangelol E (**75**) exhibited the most potent inhibitory activities against PL^pro^ and 3CL^pro^ with IC_50_ values of 1.20 and 11.40 μM (Park et al., 2016). Moreover, 10 polyphenols from *Broussonetia papyrifera* (L.) L’Hér. ex Vent. and four natural products, namely, isoliquiritigenin (**89**), kaempferol (**56**), quercetin (**22**), and quercetin-β-galactoside (**6**), were identified as inhibitors against both PL^pro^ and 3CL^pro^ of SARS-CoV or MERS-CoV. Similar to their previous studies, all bioactive molecules were more potent against PL^pro^ than 3CL^pro^. The most potent inhibitor was papyriflavonol A (**82**), which presented anti-SARS-CoV PL^pro^ activity with an IC_50_ of 3.70 μM (Park et al., 2017). In addition to these excellent studies of this team, Chen et al. recently reported ginkgolic acid (**90**) and anacardic acid (**91**) as potent covalent inhibitors of both PL^pro^ and 3CL^pro^ of SARS-CoV-2, and the two compounds showed inhibitory activities against SARS-CoV-2 replication *in vitro* at non-toxic concentrations (Chen et al., 2021).

Some other studies only reported on natural bioactive molecules that inhibited PL^pro^ (Figure 3C). Six cinnamic amides derived from *Tribulus terrestris* L. fruits exhibited inhibitory activities against SARS-CoV PL^pro^, of which terrestrimine (**97**) was the most potent inhibitor with an IC_50_ of 15.80 μM (Song et al., 2014). Cho et al. isolated 12 compounds from *Paulownia tomentosa* (Thunb.) Steud. fruits, including 5 novel geranylated flavonoid derivatives containing an unusual 3,4-dihydro-2*H*-pyran moiety. All derived components dose-dependently inhibited PL^pro^ with an IC_50_ range of 5.00–14.40 μM, and the 3,4-dihydro-2*H*-pyran moiety allowed them to inhibit PL^pro^ more strongly, especially tomentin E (**102**) with an IC_50_ of 5.00 μM (Cho et al., 2013). Moreover, six aromatic compounds from *Psoralea corylifolia* (L.) seeds were identified as potent inhibitors against SARS-CoV PL^pro^. Of these bioactive molecules, isobavachalcone (**112**, IC_50_ = 7.30 μM) and psoralidin (**114**, IC_50_ = 4.2 μM) were the two most promising compounds that inhibit PL^pro^ by reversible mixed type I mechanisms, which meant that the compounds preferred to interact with the free enzyme as opposed to the enzyme-substrate complex (Kim et al., 2014).

#### Replicase-Transcriptase Complex Proteins

As previously described, the RTC plays a dominant role in generating new genomic and sgRNAs, which are responsible for synthesizing various components of new viruses. RdRp is the core component of RTC and has been considered as an attractive drug target. Despite the development of several well-known drug molecules, such as remdesivir, ribavirin, and favipiravir, as RdRp inhibitors, a few studies have reported natural biomolecular inhibitors against RdRp (**III**, Table 1). Fung et al. reported a randomized, double-blind, placebo-controlled clinical trial result of a Chinese herbal formula named Kwan Du Bu Fei Dang (KDBFD), thought to be a potent anti-SARS-CoV agent. Further, they determined the anti-RdRp activities of KDBFD extract (**116**) and the extracts of other four traditional Chinese medicines, namely, *Houttuynia cordata* Thunb. extract (**117**), *Ganoderma lucidum* (Leyss. ex Fr.) Karst. extract (**118**), *Coriolus versicolor* (L. ex Fr.) Quel. extract (**119**), and *Sinomenium acutum* (Thunb.) Rehder & E. H. Wilson extract (**120**). The research indicated that these extracts all inhibited SARS-CoV RdRp in a dose-dependent manner with IC_50_ values ranging between 41.90 and 471.30 μg/ml (Fung et al., 2011).

**FIGURE 4:**
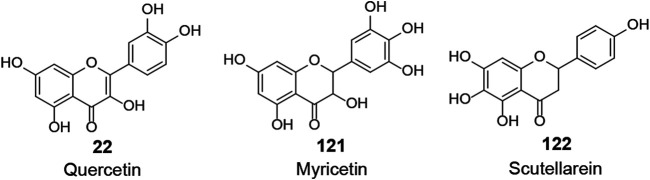
Chemical structure of different natural compounds targeting viral helicase (**IV**).

NTPase/helicase is also essential for viral replication and represents a potential target against coronaviruses. Several flavonoids were determined as inhibitors of NTPase/helicase (**IV**, Figure 4). Quercetin (**22**) was reported in several studies as an effective anti-SARS-CoV agent, and, as previously mentioned, it showed potent inhibitory activities against several targets of interest. Lee et al. investigated aryl diketoacids and its bioisostere dihydroxychromone derivatives to reveal the structure activity relationship of such compounds to selectively inhibit the duplex DNA-unwinding activity of SARS-CoV NTPase/helicase. In their study, quercetin (**22**, IC_50_ = 8.10 μM) was indicated to selectively inhibit the duplex DNA-unwinding activity in the micromolar range (Lee et al., 2009a; Lee et al., 2009b). What is more, this team introduced arylmethyl substituent at the 7-OH position of quercetin by chemical synthesis, resulting in a significant increase in inhibitory activity against SARS-CoV helicase. Of these, 4-ClPhCH_2_, 3-ClPhCH_2_, and 3-CNPhCH_2_ derivatives exhibited inhibitory activity against helicase with an IC_50_ range of 2.70–5.20 μM (Park et al., 2012a). However, another two flavonoids, myricetin (**121**) and scutellarein (**122**), were also reported to inhibit SARS-CoV Nsp13 by affecting its ATPase activity, not the unwinding activity, with IC_50_ values of 2.71 and 0.86 μM, respectively.

#### Structural Proteins

**FIGURE 5:**
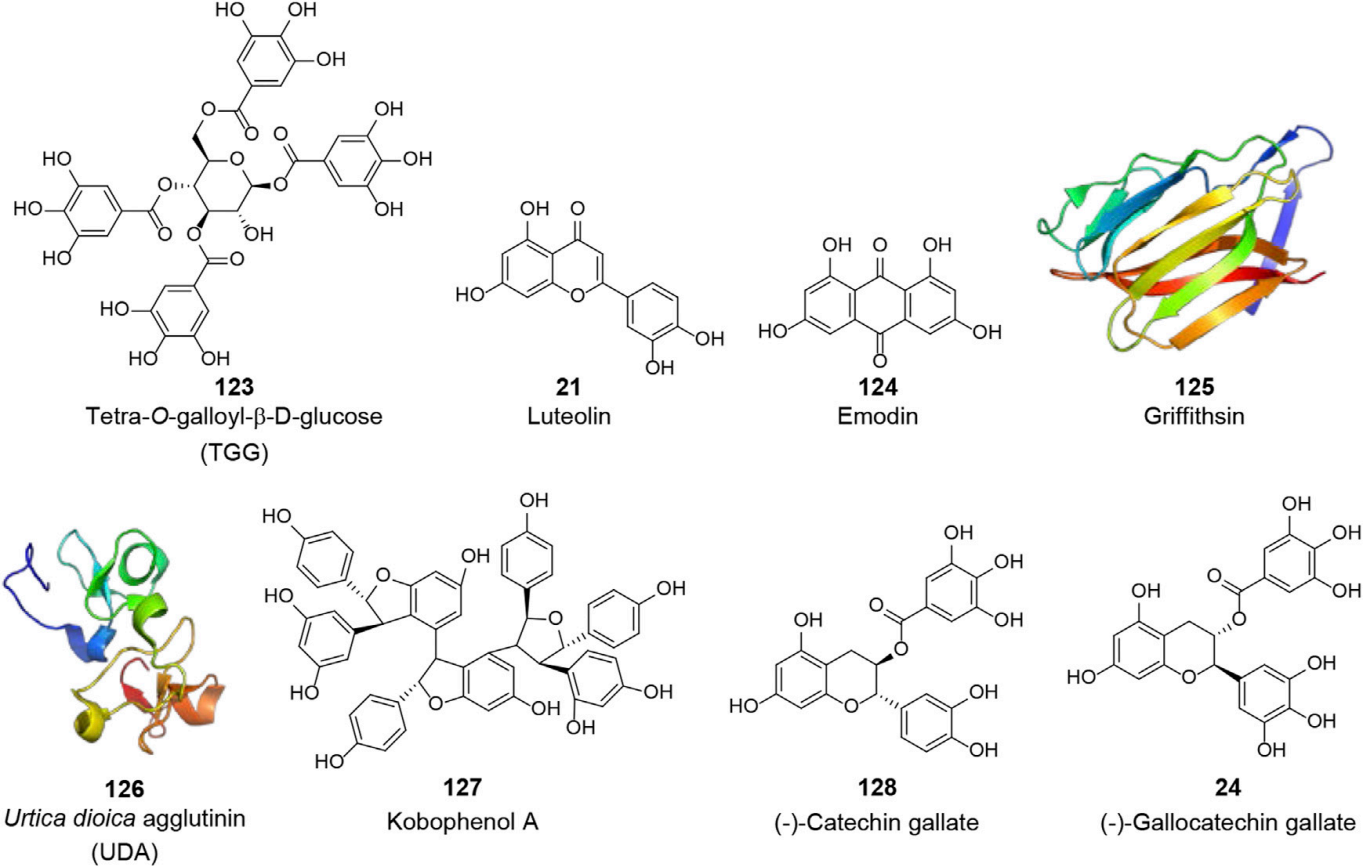
Chemical structure of different natural compounds targeting structural proteins. The S protein (**V**): tetra-*O*-galloyl-β-d-glucose (TGG, **123**), luteolin (**21**), emodin (**124**), griffithsin (**125**, PDB: 2GTY), *Urtica dioica* L. agglutinin (UDA, **126**, PDB: 1EN2), and kobophenol A (**127**). The N protein (**VI**): (-)-catechin gallate (**128**) and (-)-gallocatechin gallate (**24**).

Structural proteins are essential for viral morphology and life activities. Among the four structural proteins, the S protein is the most prominent potential target for anti-CoV drugs, because of its crucial role in virus attachment and entry through specific binding to the cellular receptor as well as conformational changes and proteolysis. Several natural products have been reported to exhibit anti-SARS-CoV activities by inhibiting the activity of the S protein or interfering with its interaction with ACE2 (**V**, Figure 5). Using frontal affinity chromatography-mass spectrometry (FAC/MS) and pseudotyped virus infection assay, Yi et al. screened 121 herbs used in traditional Chinese medicine and identified tetra-*O*-galloyl-β-d-glucose (TGG, **123**) and luteolin (**21**), with significant affinity to the S2 protein (Asn733 to Gln1190 of the SARS-CoV S protein), as agents against SARS-CoV with EC_50_ values of 4.50 and 10.60 μM, respectively (Yi et al., 2004). Emodin (**124**), a bioactive component from *Rheum officinale* Baill. and *Polygonum multiflorum* Thunb., was reported to significantly block the binding of the S protein to ACE2 with an IC_50_ of 200.00 μM as well as inhibit the infectivity of the S protein-pseudotyped retrovirus to Vero E6 cells (Ho et al., 2007). Natural lectins are a class of proteins with specific carbohydrate-binding activity as one or more non-catalytic structural domains can bind specifically and reversibly to monosaccharides or oligosaccharides. Because of the highly glycosylation on the S protein, lectins are considered as potential anti-CoV candidates (Mitchell et al., 2017). Griffithsin (GRFT, **125**, PDB: 2GTY), a lectin isolated from the red algae *Griffithsia* sp., was identified as a broad-spectrum agent against coronaviruses such as SARS-CoV and MERS-CoV (O'Keefe et al., 2010; Millet et al., 2016). This 12.7 kDa protein was shown to possess three almost identical carbohydrate-binding domains, which allowed GRFT to bind to specific oligosaccharides on envelope glycoproteins and block viral entry (Ziolkowska et al., 2006; Ziolkowska et al., 2007). Isothermal titration calorimetry (ITC) assay showed that GRFT binds to the S protein of SARS-CoV with a stoichiometry of 3:1 and a dissociation constant (K_D_) of 24.90 nM. However, it was shown that the binding of GRFT did not interfere with the interaction between the S protein and ACE2 but inhibited *in vitro* infection of distinct strains of SARS-CoV, including Urbani, Tor-II, CuHK, and Frank strains, with EC_50_ values ranging between 48.00 and 94.00 nM (O’Keefe et al., 2010). *Urtica dioica* L. agglutinin (UDA, **126**, PDB: 1EN2), an 8.7 kDa plant monomeric lectin, was reported to inhibit the viral replication of distinct strains of SARS-CoV with an IC_50_ range of 0.60–2.60 μg/ml in Vero 76 cells. In this study, UDA was also found to inhibit SARS-CoV replication in a lethal SARS-CoV BALB/c mouse model and neutralize the virus infectivity by binding to the S protein (Kumaki et al., 2011). In addition, Kobophenol A (**127**), a bioactive molecule from *Caragana sinica* (Buc’hoz) Rehder, was recently identified as a potential inhibitor that hinders the interaction between the ACE2 and the S protein *in vitro* with an IC_50_ of 1.81 μM and inhibits the viral infection of SARS-CoV-2 in cells with an EC_50_ of 71.60 μM (Gangadevi et al., 2021).

The N protein plays a vital role in virion assembly by enveloping the entire genomic RNA and participating in viral RNA synthesis. The N protein is also a major pathological determinant in the host and is important for early virus detection and disease diagnosis. Due to its crucial role, the N protein is also considered an important anti-CoV target (**VI**, Figure 5). Using a quantum dots-conjugated RNA oligonucleotide system, which simulated the direct binding of the viral RNA to the N protein on a designed biochip, Roh et al. screened 23 polyphenolic compounds to investigate potential inhibitors of the SARS-CoV N protein. (-)-Catechin gallate (**128**) and (-)-gallocatechin gallate (**24**) were found to inhibit the N protein binding to the RNA oligonucleotide in a concentration-dependent manner at 0.005 μg/ml or more. At the 0.05 μg/ml concentration, these two compounds displayed more than 40% inhibitory activity on the designed biochip (Roh, 2012).

### Host Proteins

During SARS-CoV-2 infection of human cells, some important host proteins play critical roles, including receptor ACE2 and proteases TMPRSS2/4, CatL, furin, etc. In the drug discovery against SARS-CoV-2, targeting viral proteins may be the most direct and effective strategy. However, the fact that viruses can develop drug resistance cannot be ignored. Therefore, targeting these relevant host proteins is another viable strategy. Of course, the safety of this strategy must be carefully considered and evaluated, while it is encouraging that these host proteins have been well studied as therapeutic targets for other diseases and many of the corresponding inhibitors are already in clinical use or under investigation. In the following sections, natural bioactive molecules targeting host proteins will be displayed according to their different targets.

#### ACE2

**FIGURE 6:**
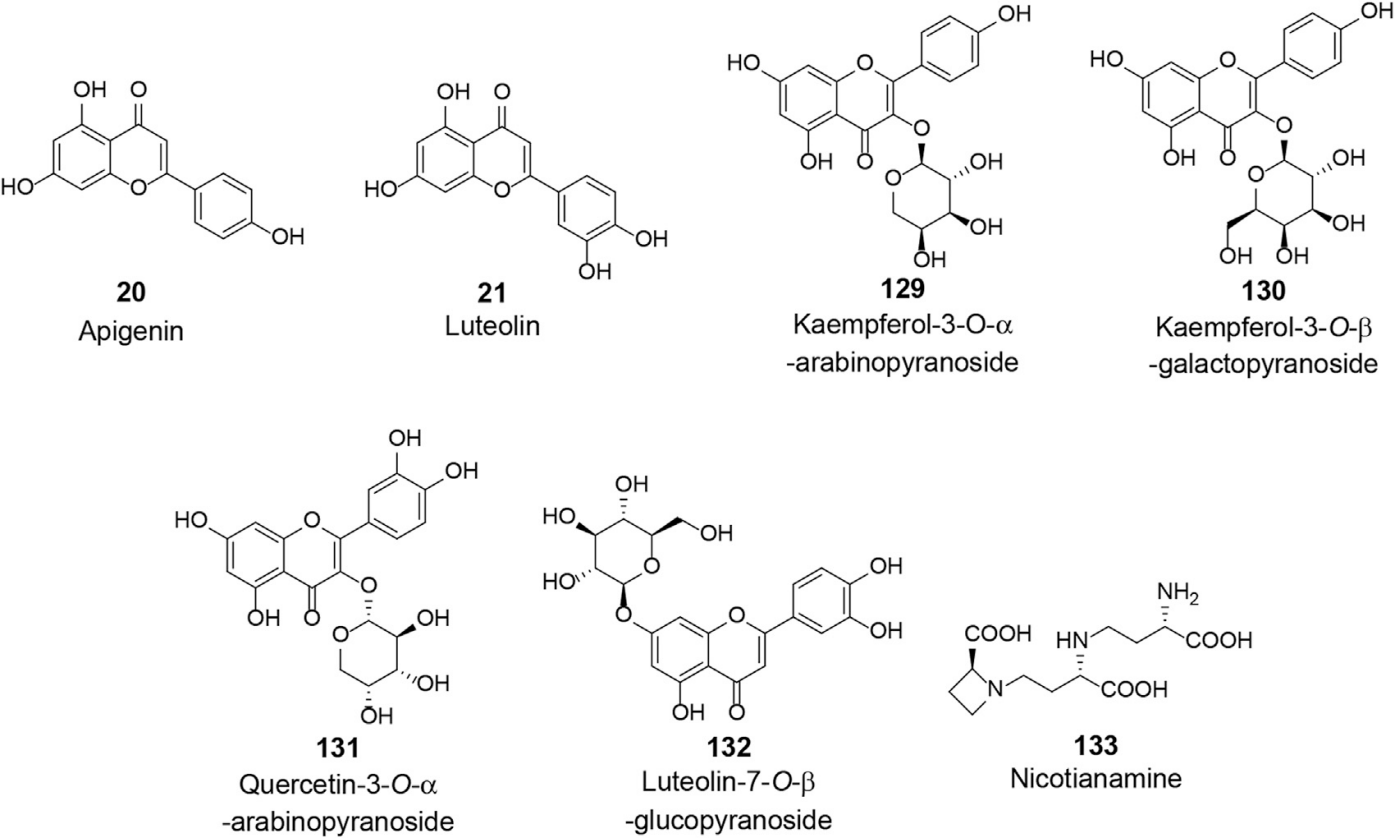
Chemical structure of different natural compounds targeting host ACE2 **(VII)**.

ACE2 is a type I integral membrane protein with a full length of 805 amino acids, including an N-terminal signal peptide sequence of 17 amino acids and a C-terminal membrane-anchored region as well as a HEXXH-E zinc-binding consensus sequence (Gheblawi et al., 2020). ACE2 has multiple roles, including the negative regulator of the renin-angiotensin system, amino acid transporter, and cellular receptor of SARS-CoV and SARS-CoV-2 (Li et al., 2003; Turner et al., 2004; Hashimoto et al., 2012; Yan et al., 2020). As previously described, after SARS-CoV or SARS-CoV-2 invades the body, the S protein binds specifically to ACE2, thus initiating the viral recognition process and entry into the host cell. As a result, drugs that could inhibit or regulate the activity of ACE2 might be potential candidates against SARS-CoV-2. An abundance of natural bioactive molecules have been reported to affect the activity of ACE2 (**VII**, Figure 6 and Table 2). Several natural products extracted from the leaves of *Ailanthus excelsa* Roxb., including apigenin (**20**), luteolin (**21**), kaempferol-3-*O*-α-arabinopyranoside (**129**), kaempferol-3-*O*-β-galactopyranoside (**130**), quercetin-3-*O*-α-arabinopyranoside (**131**), and luteolin-7-*O*-β-glucopyranoside (**132**), were identified as ACE2 inhibitors with an IC_50_ range of 260.00–320.00 μM *in vitro* using ACE2 via Elbl and Wagner methods (Loizzo et al., 2007). However, in another study, apigenin (**20**) was found to up-regulate the expression of ACE2 in the kidney in spontaneously hypertensive rats (Sui et al., 2010). Takahashi et al. synthesized various internally quenched fluorogenic substrates based on the cleavage site of ACE2 and identified Nma-His-Pro-Lys(Dnp) as the most suitable substrate that could be hydrolyzed by recombinant human ACE2. Using the recombinant human ACE2 and Dnp, nicotianamine (**133**), isolated from *Glycine max* (L.) Merr., was identified as a novel ACE2 inhibitor with an IC_50_ of 84.00 nM (Takahashi et al., 2015).

**TABLE 2:** Natural compounds targeting host proteins: ACE2 (**VII**), TMPRSS2 (**VIII**), and CatL (**IX**).

No	Compound	IC_50_	Target	References
20	Apigenin	280.00 μM	ACE2	Loizzo et al. (2007)
21	Luteolin	290.00 μM	ACE2	Loizzo et al. (2007)
129	Kaempferol-3-*O*-α-arabinopyranoside	320.00 μM	ACE2	Loizzo et al. (2007)
130	Kaempferol-3-*O*-β-galactopyranoside	260.00 μM	ACE2	Loizzo et al. (2007)
131	Quercetin-3-*O*-α-arabinopyranoside	310.00 μM	ACE2	Loizzo et al. (2007)
132	Luteolin-7-*O*-β-glucopyranoside	280.00 μM	ACE2	Loizzo et al. (2007)
133	Nicotianamine	84.00 nM	ACE2	Takahashi et al. (2015)
134	Aprotinin	No data	TMPRSS2	Shen et al. (2017)
25	Tannic acid	2.31 μM	TMPRSS2	Wang et al. (2020)
32	Celastrol	No data	TMPRSS2	(Fernandez-Quintela et al., 2020; Habtemariam et al., 2020)
135	Leupeptin	No data	Cathepsin L	(Salminen and Gottesman, 1990; Nishimura et al., 1995)
136	Gallinamide A	5.00 nM	Cathepsin L	Miller et al. (2014)
137	Panduratin A	1.50 μM	Cathepsin L	Deb Majumdar et al. (2011)
138	Nicolaidesin C	1.00 μM	Cathepsin L	Deb Majumdar et al. (2011)
139	Grassypeptolide A	14.00 μM	Cathepsin L	Kwan et al. (2014)
140	Grassypeptolide B	21.30 μM	Cathepsin L	Kwan et al. (2014)
141	Grassypeptolide C	20.40 μM	Cathepsin L	Kwan et al. (2014)

#### Host Proteases

**FIGURE 7:**
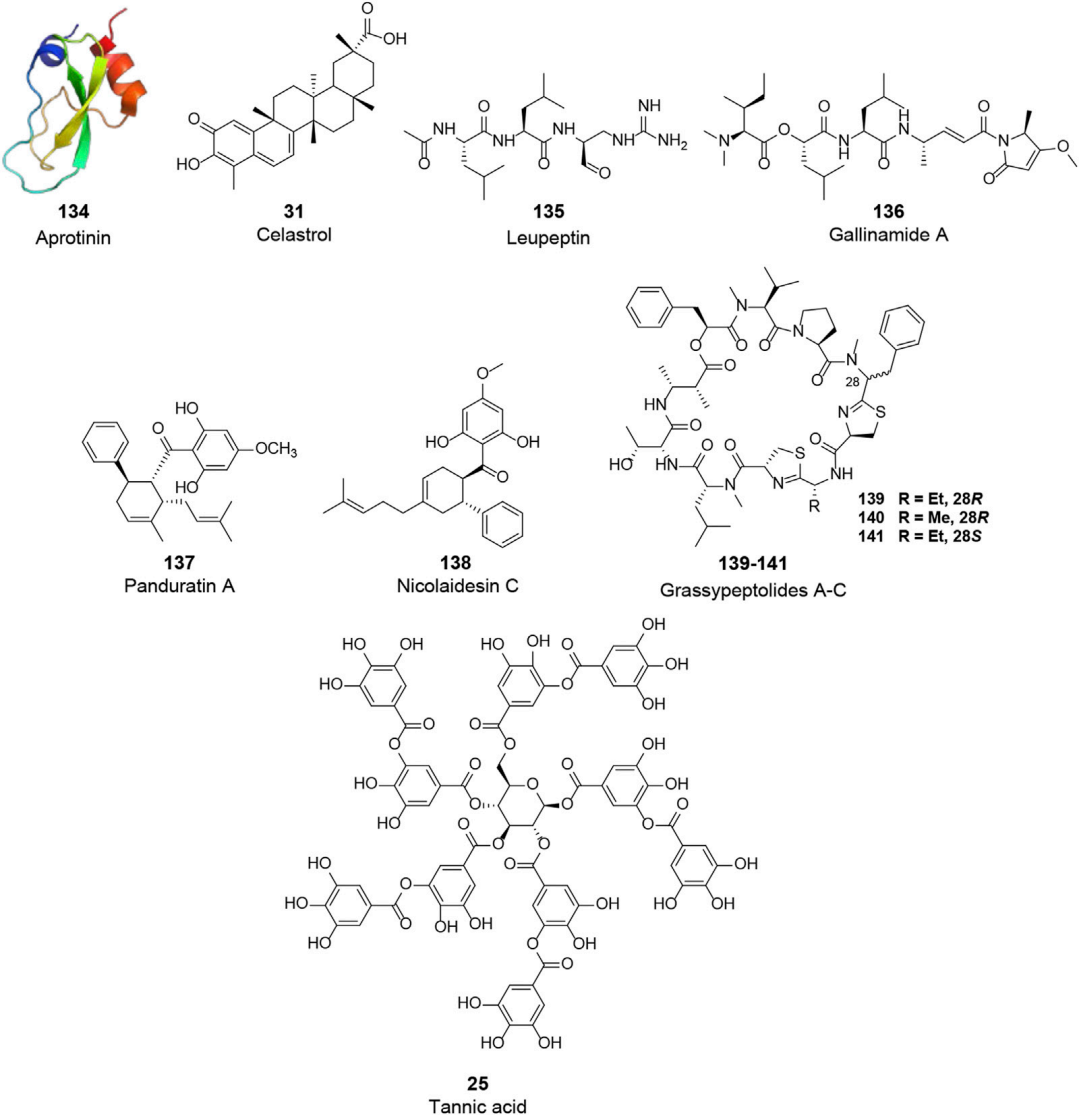
Chemical structure of different natural compounds targeting host proteases. TMPRSS2 (**VIII**): aprotinin (**123**, PDB: 1BPI), tannic acid (**25**), and celastrol (**31**). CatL (**IX**): leupeptin (**135**), gallinamide A (**136**), panduratin A (**138**), nicolaidesin C (**138**), and grassypeptolide A-C (**139–141**).

Coronaviruses have evolved multiple strategies for the S protein hydrolysis, which has been reported to be involved in various host proteases, such as TMPRSS2/4, CatL, furin, and trypsin (Millet and Whittaker, 2015). Recently, some of them have been considered potential targets for anti-CoV drugs. In the following, we will present some natural bioactive molecules that have been reported to target TMPRSS2 or CatL for their essential roles in the S protein hydrolysis **(** Figure 7; Table 2).

TMPRSS2 is type II transmembrane serine protease, which cleaves the S protein after its binding to ACE2, resulting in viral fusion to the cell membrane. Although TMPRSS2 plays an essential role, few natural molecules have been reported to inhibit the activity of TMPRSS2 (**VIII**, Figure 7). Aprotinin (**134**, PDB: 1BPI), a polypeptide consisting of 58 amino acid residues purified from bovine lung, was identified as a potential agent against TMPRSS2 (Shen et al., 2017). This polypeptide was shown to inhibit influenza virus replication by inhibiting serine proteases and suppressing the cleavage of influenza virus HA. In addition, it was shown to be effective in mice and human patients and has been approved in Russia as an aerosol for the treatment of patients with mild influenza infections (Ovcharenko and Zhirnov, 1994; Zhirnov et al., 2011). However, more studies are needed to prove its therapeutic activity in coronavirus infections. Tannic acid (**25**), with inhibitory activities against 3CL^pro^ of both SARS-CoV and SARS-CoV-2 as mentioned above, was recently reported to bind to TMPRSS2 with a K_D_ of 1.77 μM and dose-dependently inhibit TMPRSS2 activity with an IC_50_ of 2.31 μM (Wang et al., 2020). Thus, tannic acid has the promising potential to be a dual inhibitor against SARS-CoV-2. Similarly, celastrol (**34**), a 3CL^pro^ inhibitor, was found to inhibit TMPRSS2 activity. Considering its anti-inflammatory activity by suppressing NF-κB signaling, celastrol was recently suggested to be a promising drug for the treatment of COVID-19 (Wei and Wang, 2017; Shi et al., 2018; Fernandez-Quintela et al., 2020; Habtemariam et al., 2020).

In addition to TMPRSS2, an endosomal cysteine protease CatL can also hydrolyze and initiate the S protein activity, allowing the viral membrane fusion via endocytosis. Although CatL is considered dispensable for viral spread and pathogenesis in the infected host compared to TMPRSS2, a variety of natural products have been reported to inhibit this protease and are potential candidates for the treatment of COVID-19 (**IX**, Figure 7). A pulse-chase experiment in primary cultures of rat hepatocytes showed that the intracellular processing of CatL consisted of two main steps: synthesis of the 39 kDa proenzyme and maturation of the enzyme, in which the 39 kDa proenzyme was processed into 30 and 25 kDa active mature forms of CatL. Leupeptin (**135**), a non-covalent inhibitor of CatL reported by several early studies, could inhibit the maturation of CatL and lead to intracellular accumulation of the 39 kDa proenzyme (Salminen and Gottesman, 1990; Nishimura et al., 1995). Gallinamide A (**136**), isolated from cyanobacterium *Schizothrix sp.*, is the most active natural CatL inhibitor reported to date. This bioactive molecule selectively and irreversibly inhibited CatL with an IC_50_ value of 5.00 nM (Miller et al., 2014). Panduratin A (**137**) and nicolaidesin C (**138**), two cyclohexenyl chalcone Diels–Alder natural products, were identified as potential CatL inhibitors with IC_50_ values of 1.50 and 1.00 μM, respectively (Deb Majumdar et al., 2011). Notably, in a recent high-content screening of Thai medicinal plants, panduratin A was identified as an agent against SARS-CoV-2 with an IC_50_ of 0.81 μM and exhibited 99.9% inhibitory activities against SARS-CoV-2 at 10.00 μM (Kanjanasirirat et al., 2020). Although the antiviral mechanism of panduratin A was not fully revealed, its inhibitory activity of CatL might explain this observation. In addition, Kwan et al. investigated the inhibitory activities of several natural products against proteases. Three bis-thiazoline containing cyclic depsipeptides from *Lyngbya confervoides*, grassypeptolides A–C (**139–141**), were shown to inhibit several proteases, of which these compounds inhibited CatL with IC_50_ values of 14.00, 21.30, and 20.40 μM, respectively (Kwan et al., 2014).

### Unknown Targets

**FIGURE 8:**
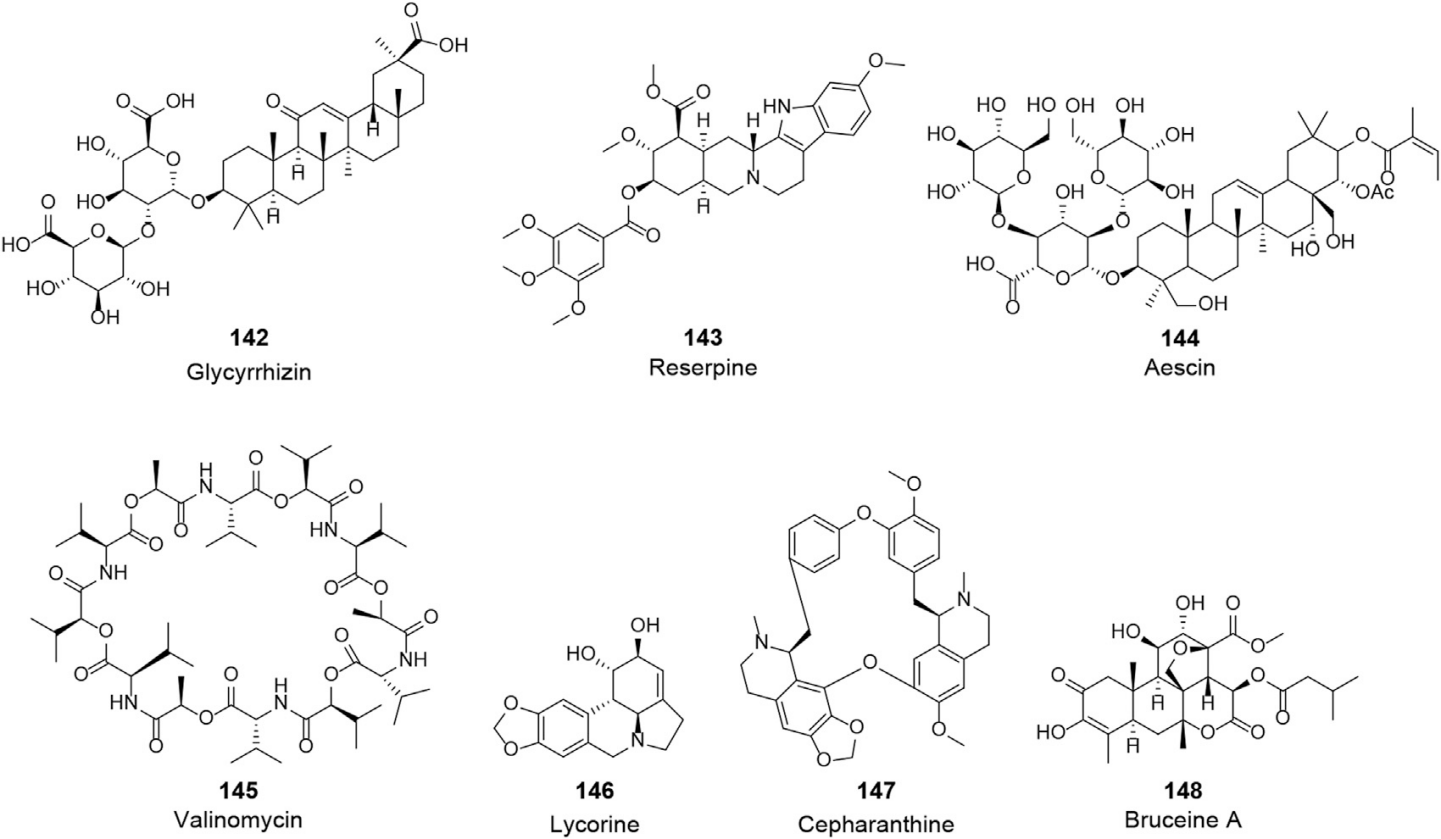
Chemical structure of different natural compounds with significant antiviral activity and without clear targets and mechanisms.

In search of anti-CoV agents, many natural bioactive molecules with unknown targets have been reported (**X**, Figure 8 and Table 3). Such natural products, which will be partially but not exclusively listed in this section, possess significant anti-CoV activity with unclear targets and mechanisms. Although more studies are needed to unravel their mechanisms, they remain worthy candidates for the treatment of COVID-19. As the primary effective extract of the well-known phytomedicine liquorice, the antiviral activity of glycyrrhizin (**142**) has been widely reported (Li et al., 2014). The study of Cinatl et al. showed that glycyrrhizin exhibited anti-SARS-CoV activity by inhibiting the viral adsorption, penetration, and replication, and it was more effective when used after the viral adsorption and exhibited the most effective inhibitory activity (EC_50_ of 300 mg/L) when given both during and after the adsorption period (Cinatl et al., 2003). Furthermore, recent studies attempted to explain the antiviral mechanism of glycyrrhizin through pharmacological analysis and *in silico* methods and suggested a variety of possibilities, including binding to ACE2, downregulating proinflammatory cytokines, inhibiting the accumulation of intracellular reactive oxygen species (ROS), inhibiting thrombin, inhibiting the hyperproduction of airway exudates, and inducing endogenous interferon. Although still insufficient to reveal the exact mechanism, glycyrrhizin may remain a potentially effective agent for the treatment of COVID-19 (Bailly and Vergoten, 2020; Luo et al., 2020; Muhseen et al., 2020). Three widely used clinical natural drugs, reserpine (**143**), aescin (**144**), and valinomycin (**145**), derived from *Rauvolfia serpentina* (L.) Benth. ex Kurz, *Aesculus hippocastanum* L., and *Streptomyces spp.*, respectively, were reported to inhibit SARS-CoV at micromolar concentration levels (Wu et al., 2004). Considering their excellent bioavailability and safety profile, these clinically approved drugs may be expected to be used directly for COVID-19 treatment. Lycorine (**146**), an alkaloid from the plants of Amaryllidaceae family, was an outstanding agent against SARS-CoV replication with an EC_50_ of 15.70 nM in a large *in vitro* screening and was also proven to inhibit SARS-CoV-2 (EC_50_ = 0.31 μM) in Vero E6 cells (Li et al., 2005; Zhang Y. -N. et al., 2020). Lycorine was reported to effectively inhibit several human coronaviruses, including HCoV-OC43, MERS-CoV, and HCoV-NL63, which suggested that lycorine might be a potent agent against coronaviruses (Shen et al., 2019). Recently, several screenings of natural products for anti-SARS-CoV-2 have been published. A recent study reported several clinically approved drugs as promising candidates for the treatment of COVID-19, including an alkaloid from the root of *Stephania japonica* (Thunb.) Miers, cepharanthine (CEP, **147**), which is clinically used for leukopenia treatment. The study suggested that CEP could be a wide-spectrum inhibitor of pan-betacoronavirus (Fan et al., 2020). Another cell-based large-scale screening identified 30 natural hits exhibiting suitable anti-SARS-CoV-2 activities with EC_50_ values ranging between 0.011 and 11.03 µM. Among these hits, quassinoid derivative bruceine A (**148**) was the most potent agent with an EC_50_ of 0.011 µM (Zhang Z.-R. et al., 2020). All of the above natural bioactive molecules have significant anti-CoV activity, and further investigation of their target proteins and mechanisms is of great significance for the development of anti-CoV drugs.

**TABLE 3:** Natural compounds with significant anti-CoV activity and without clear targets and mechanisms (**X**).

No	Viral strain	Compound	EC_50_	References
142	SARS-CoV	Glycyrrhizin	300.00 mg/L	Cinatl et al. (2003)
143	SARS-CoV	Reserpine	3.40 μM	Wu et al. (2004)
144	SARS-CoV	Aescin	6.00 μM	Wu et al. (2004)
145	SARS-CoV	Valinomycin	0.85 μM	Wu et al. (2004)
146	SARS-CoV	Lycorine	15.70 nM	Li et al. (2005)
146	SARS-CoV-2	Lycorine	0.31 μM	Zhang Y.-N. et al. (2020)
147	SARS-CoV-2	Cepharanthine	0.98 μM	Fan et al. (2020)
148	SARS-CoV-2	Bruceine A	0.011 μM	Zhang Z.-R. et al. (2020)

## Conclusion and Future Prospect

Natural products have been used as a treasure trove of drug discovery for a long time. These structurally diverse molecules exert a wide range of pharmacological activities, including outstanding antiviral activity. Considerable efforts have been devoted to the development of anti-CoV drugs from natural products, especially in the context of the challenges the world’s public health faces, such as the outbreaks of SARS-CoV in 2003 and the current SARS-CoV-2. In order to provide a more systematic understanding of the research on the anti-CoV activity of natural products, we reviewed relevant studies to date, excluding *in silico* only studies, and summarized numerous natural bioactive molecules based on their protein targets. Most of these natural products are enumerated as inhibitors against SARS-CoV and SARS-CoV-2 and a few molecules that act on MERS-CoV. Among them, flavonoids, alkaloids, terpenoids, and lectins showed encouraging anti-CoV activity, which might provide a large number of promising candidates for the development of anti-CoV drugs and offer potential weapons against SARS-CoV-2 in the present dilemma.

Nonetheless, these studies are often fragmented, and the molecules involved are essentially ubiquitous and represent only a small fraction of the structurally diverse natural products. One corresponding recommendation is to adopt high-throughput screening (HTS) and high-content screening (HCS) to systematically explore natural product resources, especially traditional natural medicines, to discover natural bioactive molecules with excellent anti-CoV activity. In addition, numerous problems still exist, such as the unclear anti-CoV mechanisms, the safety issues of natural products, and the drug resistance of coronaviruses. Technologies of structural biology, including nuclear magnetic resonance (NMR), X-ray crystal diffraction, and cryo-electron microscopy (Cryo-EM), may help to better reveal the anti-CoV mechanisms and targets of effective agents. Researchers can enhance the anti-CoV activity and the safety of natural bioactive molecules through target-based structural modifications and comprehensive safety valuation. Furthermore, to effectively fight against coronaviruses, the combination of natural agents with different targets may be a viable strategy, and the synergy between natural bioactive molecules and conventional drugs should be studied in depth.

In conclusion, there is indeed a long and winding road ahead to develop a feasible anti-CoV drug from natural bioactive lead candidates, which will predictably continue to be invested with more efforts, especially in the current SARS-CoV-2 pandemic. We hope that researchers can gain insights and valuable information from this review to aid in developing anti-CoV drugs from natural bioactive molecules.
